# A Rare Cause of Mechanical Icterus: Prostate Cancer Metastasis

**DOI:** 10.5152/tjg.2024.24508

**Published:** 2024-12-01

**Authors:** Adnan Ozkahraman, Ozan Durmaz, Yusuf Kayar

**Affiliations:** 1 Department of Internal Medicine, Van Training and Research Hospital, Van, Türkiye; 2 Division of Gastroenterology, Department of Internal Medicine, Van Training and Research Hospital, Van, Türkiye

Dear Editor,

A 71-year-old male patient was brought to the emergency department complaining of fever, yellowing of the body, and darkening of the urine. He also complained of weight loss, frequent nighttime urination, intermittent urination, and bloody urine. A physical examination revealed that the right upper quadrant of the abdomen was painful, icterus in the sclera, and prostate enlargement with hardness upon rectal examination. The laboratory tests revealed the following: total bilirubin of 6.6 mg/dL, direct bilirubin of 6.1 mg/dL, AST of 139 IU/mL, ALT of 212 IU/mL, ALP of 842 IU/mL, GGT of 1371 IU/mL, total PSA of 12 ng/mL (normal range: 0.01-3.1 ng/mL), fFree PSA of 1.44 ng/mL, free/total PSA of 8.3 (normal range >25), and CRP of 76 mg/dL. Ultrasonography revealed dilatation in the proximal and intrahepatic bile ducts, blunt ending in the distal common bile duct, and an increase in the size of the prostate with heterogeneity. Dilatation in the proximal common bile duct and intrahepatic bile ducts and blunt ending in the distal common bile duct were also confirmed in the magnetic resonance cholangiopancreatography imaging (Figure 1A). Endoscopic retrograde cholangiopancreatography (ERCP) revealed irregular infiltrative involvement around the papilla (Figure 1B). Cholangiography demonstrated dilatation in the common bile duct and intrahepatic bile ducts and blunt ending of the distal common bile duct (Figure 1C). A 10F, 10 cm Amsterdam-type plastic stent was inserted into the common bile duct after bougie dilation. Biopsy of the papilla during ERCP revealed suspicious malignant findings, but a definitive diagnosis could not be made. Repeat biopsy was performed using endoscopic ultrasound-guided fine-needle aspiration after evaluation of the periampullary region sonographically, which showed a heterogenous hypoechoic lesion with irregular borders (Figure 1D). Fine-needle aspiration was performed from this area using a 22 G needle, and the obtained sample was then spread for analysis. Subsequently, the patient underwent a prostate biopsy, which revealed prostate adenocarcinoma. The biopsy obtained from the periampullary region was interpreted as adenocarcinoma metastasis. The tumor consisted of glandular structures and cells with prominent eosinophilic cytoplasm with nucleoli forming solid groups and glandular structures (hematoxylin and eosin (H&E) ×100) (Figure 2A and  B). Positive immunohistochemical staining for NKX3.1 and AMACR was observed in the tumor, while negative results were obtained for CK7 and CK19 (Figure 2C and D). The patient was consulted with medical oncology for further treatment.

About 3.8% of all male cancer-related deaths are attributable to prostate cancer, which is the second most frequent cancer in the world among men. Prostate cancer incidence rises quickly with age, peaking between 75 and 79 years old (751 cases per 100 000).^1^ Up to 17% of patients are reported to experience metastasis, leading to higher mortality rates, despite the majority of new diagnoses being localized. It is widely acknowledged that, aside from regional lymph nodes, the skeletal system is the most frequently affected site for metastasis. It appears that some men with metastatic prostate cancer encounter unusual metastases in locations besides the bone and regional lymph nodes.^2,3^ The papilla mostly contains primary tumors, although there are few instances of secondary tumors affecting this organ. According to studies, lung, gastrointestinal tract, hematologic, and renal malignancies are the most prevalent tumors to spread to the papilla. Although metastases originating from the urogenital system are rarely reported in the literature, papilla metastases originating from the prostate have not been reported. So prostate should be added to the list of metastatic papillary lesions.^4^

Endoscopic retrograde cholangiopancreatography is a safe and effective method in the palliation of pathologies that cause mechanical icterus.^5,6^

Immunohistochemical staining is very useful in the diagnosis of adenocarcinoma metastases. Although AMACR staining has high specificity and sensitivity for prostate cancers, it may be positive in around 8% of pancreatic adenocarcinoma metastases. On the other hand, while CK7 and CK19 staining is not observed in prostate cancer, they are typically positive in approximately 100% of pancreatic carcinomas. Recent studies have reported that NKX3.1, a tumor suppressor gene located on chromosome 8p, is distinctive for prostate cancer. Likewise, the sensitivity of NKX3.1 in identifying metastatic prostate cancer was found to be 98.6% and specificity was 99.7%.^7^ In conclusion, prostate cancer is a rare cause of metastatic lesions of the papilla. However, in patients exhibiting compatible symptoms, it ought to be taken into account while making a differential diagnosis of mechanical icterus.

## Figures and Tables

**Figure 1.**
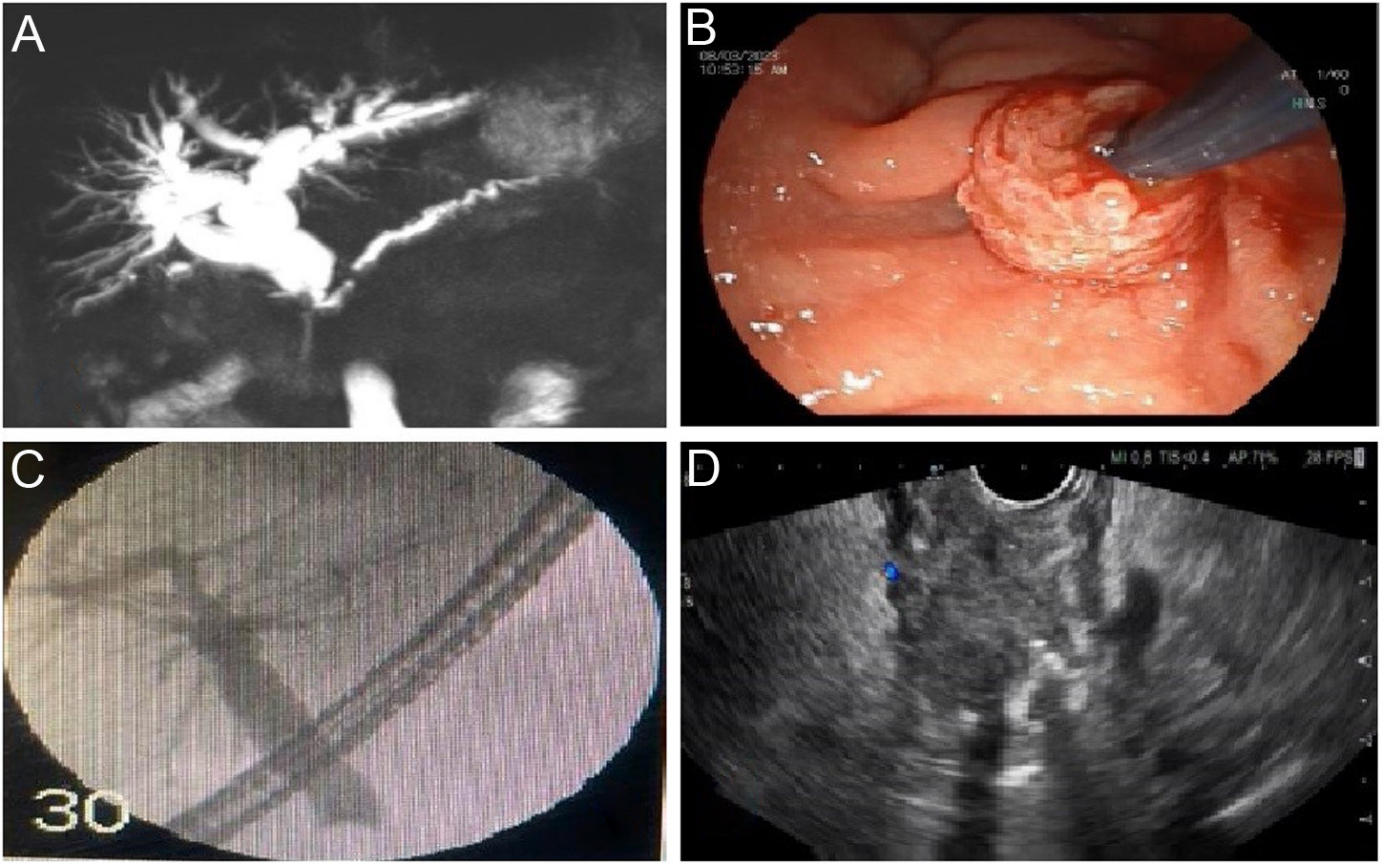
(A) Dilatation in the proximal common bile duct and intrahepatic bile ducts, with a blunt ending in the distal common bile duct as shown in magnetic resonance cholangiopancreatography imaging. (B) Irregular infiltrative involvement around the papilla in endoscopic retrograde cholangiopancreatography. (C) Cholangiography demonstrated dilatation in the common bile duct and intrahepatic bile ducts and blunt ending of the distal common bile duct. (D) Heterogenous hypoechoic lesion with irregular borders in endoscopıc ultrasonography.

**Figure 2.**
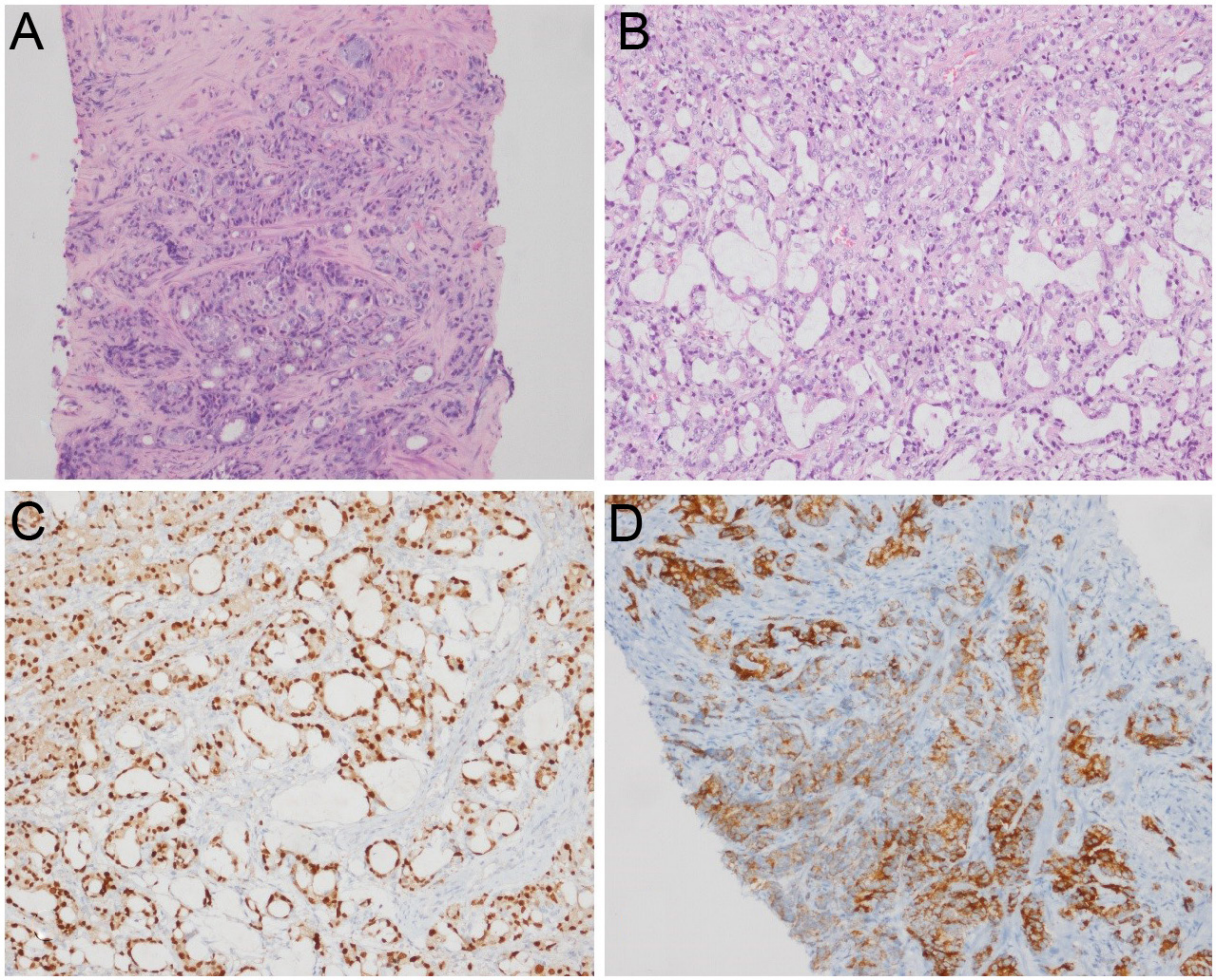
(A and B) Tumor consisted of glandular structures and cells with prominent eosinophilic cytoplasm with nucleoli forming solid groups and prominent nucleoli forming solid groups, and glandular structures (H&E ×100). (C and D) Positive immunohistochemical staining for NKX3.1 and AMACR was observed in the tumor.

## References

[1] Gandaglia G Abdollah F Schiffmann J , et al. Distribution of metastatic sites in patients with prostate cancer: a population-based analysis. Prostate. 2014;74(2):210 216. (10.1002/pros.22742) 24132735

[2] Scosyrev E Messing EM Mohile S Golijanin D Wu G . Prostate cancer in the elderly: frequency of advanced disease at presentation and disease-specific mortality. Cancer. 2012;118(12):3062 3070. (10.1002/cncr.26392) 22006014

[3] Hess KR Varadhachary GR Taylor SH , et al. Metastatic patterns in adenocarcinoma. Cancer. 2006;106(7):1624 1633. (10.1002/cncr.21778) 16518827

[4] Adsay NV Andea A Basturk O Kilinc N Nassar H Cheng JD . Secondary tumors of the pancreas: an analysis of a surgical and autopsy database and review of the literature. Virchows Arch. 2004;444(6):527 535. (10.1007/s00428-004-0987-3) 15057558

[5] Karakaya MF Er E Kırımker O , et al. Management of biliary complications in liver transplant recipients with duct-to-duct anastomosis: a single-center experience. Turk J Gastroenterol. 2023;34(2):177 181. (10.5152/tjg.2023.22724) 36843302 PMC10081117

[6] Cao D Li H Wang J Zhang F Zhao H Ren C . Oral endothelium corneum Gigeriae Galli therapy for pancreatic duct stones: a prospective cohort study. Turk J Gastroenterol. 2022;33(12):1050 1057. (10.5152/tjg.2022.22086) 36098361 PMC9797788

[7] Gurel B Ali TZ Montgomery EA , et al. NKX3.1 as a marker of prostatic origin in metastatic tumors. Am J Surg Pathol. 2010;34(8):1097 1105. (10.1097/PAS.0b013e3181e6cbf3) 20588175 PMC3072223

